# The utilization and management of plant resources in rural areas of the Limpopo Province, South Africa

**DOI:** 10.1186/1746-4269-9-27

**Published:** 2013-04-16

**Authors:** Marula T Rasethe, Sebua S Semenya, Martin J Potgieter, Alfred Maroyi

**Affiliations:** 1 Department of Biodiversity, University of Limpopo, Private Bag X1106, Sovenga 0727, South Africa

**Keywords:** Conservation, Management, Plant resources, Limpopo Province, South Africa

## Abstract

**Background:**

Most rural people in the Limpopo Province depend on plant resources to meet their livelihood needs. However, there is insufficient recorded information regarding their use and management. The current study therefore was carried out in selected villages of the Limpopo Province, to close this knowledge gap.

**Methods:**

Information was collected from 60 people residing in two villages, using a semi-structured questionnaire, supplemented with field observations.

**Results:**

A total of 47 wild plant species (95% indigenous and 5% exotics) from 27 families, mostly from the Fabaceae (17%), Anacardiaceae (9%), and Combretaceae (9%) were documented. These species were used primarily for firewood (40%), food (36%) and medicine (29%). Significantly used species included *Sclerocarya birrea* (85%), *Combretum kraussii* (35%) and *Harpephyllum caffrum* (35%). Local traditional rules and regulations including taboos, social beliefs and fines are in place to aid in the management of communal resources. However, a significant number (67%) of participants mentioned that they were not pleased with these rules and regulations.

**Conclusion:**

The current study concluded that plant resources still play an important role in the surveyed rural areas of the Limpopo Province. Furthermore, for sustainable utilization and long-term conservation of plants in these areas the government should assist communities in the management of their plant resources.

## Background

Since time immemorial, Africans have gathered plant resources to meet their livelihood needs [1]. These resources include amongst others food, fodder, construction material, and fibres for clothing [2]. This heavy dependency on plant resources is largely conditioned by various factors that include their accessibility and socio-cultural value [3]. Even today, studies [4-10] have noted the dependency of a large part of the African population on gathered plant resources [11].

Africans in the rural Limpopo Province of South Africa are still very dependent on their local environment to meet their daily livelihood needs [12]. However, there is still a dearth of recorded information about communal use of plant resources, as well as its management by, and perceptions of adopted management strategies by local communities. According to Ramakrishnan [13], local people’s perceptions of, plant biodiversity and its management influence the type of interactions they have with their surroundings, which ultimately play an important role in local conservation efforts [14].

Therefore, the aim of this study was to document the use of plant resources by people in two rural villages of the Limpopo Province, and explore perceptions towards adopted management of these resources. Findings of this study will assist to define the “plant-people relationship” that is important for the ultimate sustainable utilization of plant resources in the Limpopo Province.

## Methods

### Study area

**Figure 1:**
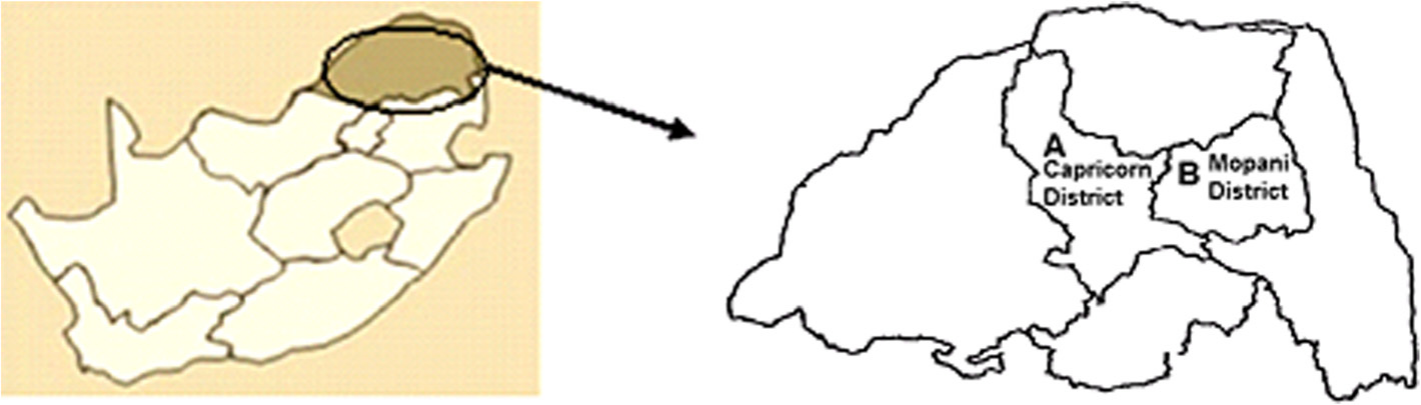
Localities of (A) Monywaneng and (B) Ga-Sekgopo villages in the Limpopo Province, South Africa.

The study was conducted in two villages situated in the Capricorn (Monywaneng) and Mopani (Ga-Sekgopo) districts of the Limpopo Province. Monywaneng village is situated 30 km north-west of the city of Polokwane, and Ga-Sekgopo 80 km north east of Polokwane (Figure 1). These two villages were selected as representatives of both peri-urban (Monywaneng) and rural (Ga-Sekgopo), thus covering the socio-economic spectrum of communities that rely on their surrounding vegetation for their livelihood.

The interviewed people in the present study are from the Bapedi ethnic group. According to Monning [15], this ethnic group depend on natural plant resources to meet their different needs such as food, furniture, fuelwood and fibre. Semenya et al. [16], noted that Bapedi people are heavily reliant the medicinal plants, either alone or in combination, with orthodox medicines for the treatment of several diseases.

### Description of vegetation types

The vegetation in the studied areas is classified as semi-arid savannas [17], characterized by a mixture of trees, shrubs and grasses [18]. This type of vegetation provides a diverse flora with rich useful plant species that the people of the study areas utilise to meet their livelihood needs. Dominant tree genera include *Acacia*, *Albizia*, *Combretum*, *Gymnosporia*, *Grewia*, *Sclerocarya* and *Terminalia*.

### Climate

Generally, the studied villages; Ga-Sekgopo and Monywaneng have a semi-arid type of climate [19]. They are located in a summer rainfall region where the rainfall occurs in the form of heavy thunderstorms or soft rain. These villages receive almost 90% of their total annual rainfall during the summer period, from October to March [20].

Regarding the daily temperatures in the studied villages; they vary from mid-20's to mid-30's, with an average range of between 17° and 27°C in the summer and 4° to 20°C in the winter [20]. Furthermore, hail and frost rarely occurs.

### Sampling and data collection

Thirty adults of various ages, but older than 21, were randomly selected per village to participate in this study. Sepedi, the local dialect, was used to inform participants about the nature of the study. Prior to data collection verbal consent was obtained from each participant. Ethical conduct was insured by following the principles contained in the Code of Ethics of the University of Limpopo, and International Society of Ethnobiology [21].

Subsequently, data was collected (January to June 2012) using a semi-structured questionnaire. The questionnaire was designed to gather data on various aspects relating to the use of plant resources and people’s perception towards current vegetation management strategies in their local areas. For instance, aspects relating to plant utilization (different uses of plant resources, source of plant and harvesting frequency of plants), and those relating to adopted local management for plants (current management adopted in the studied villages, participant’s perceptions/attitudes or opinion on current management practices), and other related information were documented.

### Specimen collection and personal observations

After interviewing participants, they escorted the researcher to the field for plant identifications. Plants were initially identified by participants with their vernacular names. Collected specimens were later validated at the Larry Leach Herbarium (UNIN) of the University of Limpopo. Collection numbers of species are presented in Table 1.

**Table 1:** Useful species used by people in Ga-Sekgopo and Monywaneng villages

**Species name**	**Collection no**	**Vernacular name**	**Family**	**Utilization category**	**Ga-Sekgopo**	**Monywaneng**	**Frequency (%)**
*Acacia karroo* Hayne	43 MT	Mushu	Fabaceae	Firewood	-	+	22
*Acacia rehmanniana* Schinz	20 MT	Mosibihla	Fabaceae	Firewood	-	+	15
*Aloe greatheadii* Schönland var. *greatheadii*	16 MT	Sekgopha	Aloaceae	Medicine	+	+	22
*Asparagus suaveolens* Burch.	04 MT	Sephatlalatsa	Asparagaceae	Medicine	+	-	08
*Bauhinia galpinii* N.E.Br	28 MT	Motswiriri	Fabaceae	Medicine	-	+	05
*Berchemia discolor* (Klotzsch) Hemsl.	34 MT	Mogwahlapa	Rhamnaceae	Firewood	+	-	35
*Burkea africana* Hook.	42 MT	Monatlo	Fabaceae	Firewood	+	-	17
				Medicine	+	-	05
*Carissa edulis* (Forssk.) Vahl	36 MT	Mothokolo	Apocynaceae	Firewood	-	+	02
				Fruit	+	-	10
*Combretum apiculatum* Sond. subsp. *Apiculatum*	19 MT	Khuditshwane	Combretaceae	Firewood	+	-	07
*Combretum hereroense* Schinz	22 MT	Mokabe	Combretaceae	Firewood	+	-	02
*Combretum kraussii* Hochst.	11 MT	Moduba	Combretaceae	Firewood	+	+	35
*Clerodendrum glabrum* E.Mey.	18 MT	Mohlokohloko	Verbenaceae	Crafting	+	-	03
				Timber	+	-	02
*Commiphora mollis* (Oliv.) Engl.	32 MT	Mokgorokgoro	Burseraceae	Crafting	-	+	02
*Cryptocarya* sp.	47 MT	Morobarobe	Lauraceae	Medicine	-	+	07
*Dichrostachys cinerea* (L.) Wight & Arn.	12 MT	Moretshe	Fabaceae	Firewood	+	+	22
*Diospyros lycioides* Desf. subsp. *Lycioides*	17 MT	Setlommana	Ebenaceae	Fruit	+	-	07
*Dombeya rotundifolia* (Hochst.) Planch. var. *rotundifolia*	01 MT	Mogokobu	Sterculiaceae	Firewood	-	+	10
*Ehretia rigida* (Thunb.) Druce subsp. *Rigida*	25 MT	Morobe	Boraginaceae	Fruit	-	+	13
*Eucalyptus grandis* W.Hill ex Maiden	15 MT	Motlouma	Myrtaceae	Firewood	+	-	02
*Euclea crispa* Gürke subsp. *crispa*	33 MT	Mkwerekwere	Ebenaceae	Crafting	-	+	03
*Euclea undulate* Thunb.	46 MT	Mohlakola	Ebenaceae	Fruit	-	+	10
*Ficus ingens* (Miq.) Miq.	08 MT	Monokane	Moraceae	Fruit	+	-	12
*Flacourtia indica* (Burm.f.) Merr.	38 MT	Morethema	Flacourtiaceae	Fruit	+	-	02
*Flueggea virosa* (Roxb. ex Willd.) Voigt subsp. *Virosa*	44 MT	Mohlakauma	Phyllanthaceae	Fruit	+	+	05
				Crafting	+	-	03
*Grewia bicolor* Juss. var. *bicolor*	30 MT	Morethwa	Malvaceae	Fruit	+	+	15
*Grewia retinervis* Burret	02 MT	Mpharatshwene	Malvaceae	Firewood	+	-	05
				Medicine	+	-	02
*Gymnosporia senegalensis* (Lam.) Loes.	37 MT	Sephathwa	Celastraceae	Medicine	+	-	02
*Gymnosporia* sp.	21 MT	Moritidi	Celastraceae	Firewood	+	-	08
				Crafting	+	-	02
*Harpephyllum caffrum* Bernh. ex Krauss	27 MT	Motshidi	Anacardiaceae	Firewood	-	+	07
				Fruit	+	+	28
*Indigofera* sp.	31 MT	Morotelashotsi	Fabaceae	Medicine	-	+	05
*Kleinia longiflora* DC.	03 MT	Mmale	Asteraceae	Medicine	-	+	05
*Lannea discolor* (Sond.) Engl.	05 MT	Mokgothwane	Anacardiaceae	Fruit	-	+	05
*Lippia javanica* (Burm.f.) Spreng.	06 MT	Mosunkwane	Verbenaceae	Medicine	+	+	08
*Melia azedarach* L.	40 MT	Mobidi	Meliaceae	Firewood	-	+	10
*Opuntia ficus-indica* (L.) Mill.	41 MT	Motloro	Cactaceae	Fruit	+	+	05
*Pappea capensis* Eckl. & Zeyh.	10 MT	Morothodi	Sapindaceae	Fruit	+	-	05
*Peltophorum africanum* Sond.	45 MT	Mosehla	Leguminosae	Firewood	-	+	10
*Philenoptera violacea* (Klotzsch) Schrire	60 MT	Mphato	Leguminosae	Firewood	+	+	13
*Ptaeroxylon obliquum* (Thunb.) Radlk.	23 MT	Molope	Rutaceae	Fruit	+	-	13
*Rhigozum brevispinosum* Kuntze.	29 MT	Not available	Bignoniaceae	Medicine	-	+	02
*Sansevieria* sp.	30 MT	Mokgosi	Dracaenaceae	Crafting	+	-	02
*Sclerocarya birrea* (A.Rich.) Hochst. subsp. *caffra* (Sond.)	13 MT	Morula	Anacardiaceae	Fruit	+	+	33
				Firewood	-	+	42
				Medicine	+	+	10
*Searsia leptodictya* (Diels) T.S.Yi, A.J.Mill. & J.Wen	07 MT	Mohlohlo	Anacardiaceae	Fruit	-	+	08
*Senna petersiana* (Bolle) Lock.	26 MT	Monepenepe	Fabaceae	Medicine	+	+	05
*Solanum lycopersicum* L.	09 MT	Motamati	Solanaceae	Medicine	+	-	02
*Vangueria infausta* Burch. subsp. *Infausta*	24 MT	Mmilo	Rubiaceae	Fruit	+	+	17
*Ziziphus mucronata* Willd. subsp. *Mucronata*	14 MT	Mokgalo	Rhamnaceae	Firewood	-	+	12
				Fruit	+	+	03

**KEY:** + :Species is used, **-** : Species is not used.

### Data analysis

Collected data were carefully checked by researchers for completeness and reliability. Descriptive statistics such as frequencies and percentages were used in the analysis of the data. Information obtained from the International Plant Index (IPNI) [22] was used to validate the documented species and to establish their families.

## Results and discussion

### Diversity of plants species used

Forty seven species belonging to 41 genera and 27 botanical families, were documented as being used by participants in the two surveyed villages (Table 1). With the exclusion of *Melia azedarach* and *Opuntia ficus-indica*, all the recorded species are indigenous to South Africa. Among the botanical families, the most used species came from the Fabaceae (17%), Anacardiaceae (9%), and Combretaceae (9%). This observation is in partial agreement to that of Semenya and Maroyi et al. [23] in different districts of the Limpopo Province, they noted the dominance of Anacardiaceae and Fabaceae. These families, which are mostly trees, play a vital economic role in the present study, in being used either as fuelwood, in crafting, or as medicinal species, and are thus highly preferred.

The species recorded in the studied villages were utilized by participants for firewood (40%), fruits (36%), medicine (29%), crafting purposes (12%) and timber (2%). It is worth noting that 15% of these species were multi-used. This finding is in agreement with other studies [24,25] in the Limpopo Province. Species predominantly used for firewood include *Sclerocarya birrea* (42%), *Berchemia discolor* (35%), *Combretum kraussii* (35%), *Acacia karroo* and *Dichrostachys cinerea* (22% each). With the exclusion of *S. birrea*, which is exclusively used because of its local availability*,* all these species are mainly used due to both their local availability and long burning period, which provide lasting heat and light. The extensive use of *S. birrea* and *D. cinerea* for firewood came as no surprise as it was also listed by Madubansi and Shackleton [26] as amongst the highly preferred species for firewood by people in five villages of Bushbuckridge in the Limpopo Province. These species are preferred because they have relatively dense wood that burns well with little smoke [26].

The finding that residents in the investigated villages extensively use *S. birrea* for firewood is unfortunate as this species is protected in terms of the National Forests Act of 1998 (Act 84 of 1998) of South Africa. In terms of this Act, species may not be cut, disturbed, damaged or destroyed and their products may not be possessed, collected, removed, transported, exported, donated, purchased or sold - except under license granted by the Department of Water Affairs and Forestry [27].

The most preferred fruit species include *S. birrea* (33%), *Harpephyllum caffrum* (28%), *Vangueria infausta* (17%), *Grewia bicolor* (15%), *Ehretia rigida* (13%), *Ptaeroxylon obliquum* (13%) and *Ficus ingens* (12%). According to the participants, information on the use of these species was acquired through their parents and grandparents from a very early childhood. The extensive exploitation of some of these species is not restricted to the current study. They are commonly harvested for their fruits by different ethnic groups [28-30] residing in various geographical areas of South Africa.

Results of this study further indicated that *Aloe greatheadii* (22%) and *S. birrea* (10%) are the most preferred species used for medicinal purposes. The possible efficacy of *A. greatheadii* is confirmed by Botes [31] who extracted the different compounds (organic acid, polyphenols/phenolic acid, alcohol, aldehyde, ketone, alkane, pyrimidine, indole, alkaloid, phytosterol, fatty acid, dicarboxylic acid contents and antioxidants), which are known to have various healing properties*.* The effectiveness of *Sclerocarya birrea* to serve as a medicinal plant is culturally validated through its use by various [4,32-36] southern African cultures. However, because *S. birrea* is multi-used for medicine, firewood and food, its future utilisation should proceed with caution in order to ensure long term sustainable use. In this regard communication between environmental agencies and local communities is vital.

The same is true with other of species which are multi-used. These species are *Burkea. africana* (firewood and medicine)*, Carissa edulis* (firewood and fruit)*, Clerodendrum glabrum* (crafting and timber)*, Grewia retinervis* (firewood and medicine)*, Gymnosporia* sp. (firewood and crafting)*, H. caffrum* (firewood and fruit), and *Ziziphus mucronata* (firewood and fruit). Semenya [37] noted that the multi-utilization of a single species has disadvantages from a conservation point of view because it amplifies its harvesting pressure, thereby posing a threat. This fact is supported by Shanley and Luz [38], who observed that the multi-utilization of some single forest plants for firewood, medicine, crafting and timber has resulted in their decline and extinction in the Eastern Amazonia.

### Use of species per village

The number of plant species used by the participants in the two studied villages differs considerably (Table 1). More species were recorded in Ga-Sekgopo (77%) than in Monywaneng (67%). This difference might be an indication of the value and extent of reliance on plant biodiversity between the two communities.

However, species such as *A. greatheadii*, *C. edulis*, *Combretum kraussii*, *D. cinerea*, *Flueggea virosa, Grewia bicolor*, *H. caffrum*, *Lippia javanica*, *O. ficus-indica*, *Philenoptera violacea*, *S. birrea*, *V. infausta* and *Z. mucronata* were common in both villages. Their widespread use might be linked to their distribution or natural occurrence; an aspect that warrants further investigation.

### Source of plants

Sixty percent of people in both Monywaneng and Ga-Sekgopo mentioned that they collect plants from communal areas. The most common reasons for the preferences of this land include free access to plants, especially those used for firewood. Firewood is highly used as an alternative source of energy (due to the high cost of electricity) for cooking, boiling water, and heating houses. However, participants complained about the increased distance to collection sites, and availability of certain species compared to the past. This has resulted in them harvesting material from nearby communities, thus initiating conflict with those communities. Those community members who did not collect material in communal areas cited the availability of electricity, unavailability of preferred species, and safety factors. A study by Paumgarten and Shackleton et al. [39] in various areas of South Africa, found that those villagers who did not utilise communal areas for livelihood needs tended to purchase medicine, firewood and food. This difference in the utilisation of communal lands correlates highly with the socio-economic status of participants and their proximity to major metropolitan areas*.*

### Harvesting frequency

**Figure 2:**
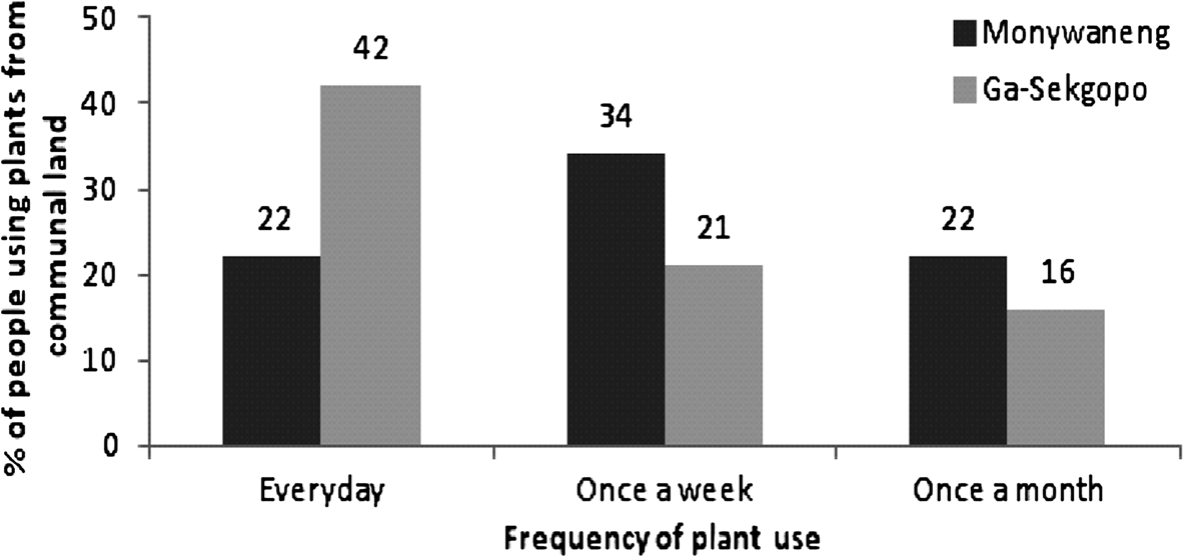
Percentages of harvesting frequencies between Monywaneng and Ga-Sekgopo villages.

Data from this study clearly indicate that a fixed protocol as to how often wild plants should be collected did not exist (Figure 2). Plants were collected throughout the year, and seasonality only played a role when harvesting fruits. However, harvesting frequency in Ga-Sekgopo village (42%) was higher than that of Monyaneng (34%). A significant number of participants in Ga-Sekgopo harvest plant material almost on a daily basis compared to those residing in Monywaneng, who collect once a week. This might be due to the difference in lifestyles between participants, mostly influenced by locality. For instance Monywaneng village is located closer to the city of Polokwane, and people in this village regularly use electricity as their primary source of energy and paraffin for cooking except during winter. In contrast Ga-Sekgopo is situated far from urban areas, and the majority of people are dependent on wood as a source of fuel for cooking and for the provision of other needs on a daily basis.

### Local management of plants

Traditional rules and regulations (Table 2) are the only adopted strategies for the management of plants in the studied villages. The most common ones were restriction of people from entering some areas of communal lands, cutting green plants, cutting trees in the graveyard, soil collection in the communal lands, harvesting of some species during certain seasons, exclusive harvesting of leaves of certain species, and collection of lateral roots from plants used medicinally. Some of these traditional rules and regulations are universal amongst different ethnic groups in African countries [9,40-45].

**Table 2:** Local traditional rules and regulations used to manage plant resources in the surveyed villages

**Traditional rule and regulations**	**Ga-sekgopo**	**Mongwaneng**
No cutting of green plant	+	+
No cutting trees in the graveyard	+	+
No soil collection in the communal land	+	+
No plant collection in times of initiation schools	+	-
Woman are not allowed to collect plants during menstruation periods	+	-
Pregnant woman are not allowed in communal lands	+	-
Some species are only harvested during certain seasons	+	-
Some species are only harvested for their leaves	+	+
Certain parts of communal lands are restricted for collection	+	+
Only small quantity of plant are collected	-	+
Only lateral roots of plants are collected	+	+
Stem bark is harvested on one side	-	+
Permit required for harvesting firewood and timber	+	-

**KEY**; + : Traditional rule and regulation used, - : Traditional rule and regulation not used.

Participants in the current study indicated that chiefs and indunas (headmen of the village) enforce the compliance rules and regulations in villages. Most participants indicated that they are restricted from entering certain areas of communal lands, as these are considered sacred, and viewed by traditional leaders as a way of preserving cultural heritage. Another control mentioned by participants was the cutting of green plants in the wild, and felling of trees in graveyards. Cutting of green branches and twigs of *P. capensis* (in the wild) was prohibited, with only harvesting of its fruits allowed. Similarly, felling of *A. karroo*, *A. rehmanniana* and *P. africanum* from the graveyard was forbidden, because graveyards are believed to be the home of the ancestors, who bring peace in the village. Thus collection of any sort of natural resource in this area will upset them, and consequently there will be no peace in the village. This cultural belief and practice is widespread in southern Africa [9,41,46], and has some positive impacts on conservation and sustainable use of resources.

This study found that the harvesting of bark for medicinal purposes is also regulated under local and traditional law. For example harvesting of stem bark of *S. birrea* is exclusively on the eastern side. This is due to the perception that bark harvested from this side of a tree contains more healing ingredients due to the belief that westerly winds carry healing powers. This traditional method of harvesting prevents trees from being ring barked and thus aid in their conservation.

Similarly, only lateral roots from medicinal species such as *P. obliquum* and *G. senegalensis*, and leaves of *L. javanica* can be harvested under customary law, a phenomenon also observed by Mabogo [4] in the Venda region of the Limpopo Province. This practice causes less impact on the survival of an individual species since the tap root is not affected. The practice of harvesting leaves for medicinal purposes are seen [35,42,47] as more sustainable because it causes less structural impact on a plant. Thus the current study affirmed that traditional rules and regulations can play an important role in the conservation and the management of wild plant resources. However, this can only happen if these rules and regulations are adhered to.

### Attitudes on traditional rules and regulations

Just over two thirds (67%) of participants noted that they are not happy with current traditional laws that are used to manage communal plant resources in their villages. They noted that these laws are not effective, simply because users of these resources do not comply with them, leading to overexploitation. Various reasons have been put forward for this non-compliance; these include, traditional leaders being inexperienced in implementing and enforcing regulations, as well as very young leaders who do not command respect. These factors have led communities to suggest that governmental agencies should play a prominent role in the decision-making process and the management of their communal plant resources. Reasons put forth by those not in favour of government-led intervention, cite that government officials mostly enforces laws that are not in harmony with their lifestyle. Furthermore, these community members felt that they are not included in the decision making process and thus feel alienated; a finding also reported by Adams [48] and Boonzaaier [49]. Thus it is clear that if government do get involved in the management of natural resources in communal lands, that this should be in an advisory capacity to empower community elders and leaders, both in terms of knowledge and status.

## Conclusion

The current study concludes that plants still play an important role in the surveyed rural areas of the Limpopo Province. Furthermore, for sustainable plant resources utilization in these areas, various state-sponsored management and conservation strategies must be combined with the use of traditional practice. This can be achieved by the establishment of community-based natural resource management mechanisms. In this mechanism, the community members and their traditional leaders are given full control on management of their plant resources in communal lands, with full support from government. For instance, government employees’ who are experts in the field of natural resource management should capacitate the community members, with relevant skills on how to manage communal lands based-natural resource; such as the sustainable methods of harvesting wild natural resource, and different strategies of natural resource management (such as domestication of some plants in home gardens, and the establishments of nature reserve for commonly used plants). Furthermore, they should educate the community members on the benefits of managing natural resource. These will, according to Moeng and Potgieter [50], enable communities to manage their environment on ecological principles and benefit economically from becoming stewards over plant biodiversity. Damn [51] noted that community conservation activities also could lead to the re-establishment of grass roots democracy and the freedom to control their destinies, which would further improve the socio-economic status of communities and by that, benefit conservation.

## Competing interests

The authors declare that they have no competing interests.

## Authors’ contributions

MTR and SSS wrote the manuscript. MJP and AM, respectively; helped to finalise manuscript. Field work was executed by MTR. All authors have read and approved the final manuscript.
